# Preparation of Acetylcholinesterase Inhibitory Peptides from Yellowfin Tuna Pancreas Using Moderate Ultrasound-Assisted Enzymatic Hydrolysis

**DOI:** 10.3390/md23020075

**Published:** 2025-02-09

**Authors:** Pai Peng, Hui Yu, Meiting Xian, Caiye Qu, Zhiqiang Guo, Shuyi Li, Zhenzhou Zhu, Juan Xiao

**Affiliations:** 1 Hainan Engineering Research Center of Aquatic Resources Efficient Utilization in South China Sea, Key Laboratory of Seafood Processing of Haikou, School of Food Science and Engineering, Hainan University, Haikou 570228, China; pengpai152@163.com (P.P.); 22110832000012@hainanu.edu.cn (H.Y.); 22220951350051@hainanu.edu.cn (M.X.); 17305393467@163.com (C.Q.); 2 School of Marine Science and Engineering, Hainan University, Haikou 570228, China; guozq@hainanu.edu.cn; 3 National R&D Center for Se-Rich Agricultural Products, Processing, Hubei Engineering Research Center for Deep Processing of Green, Se-Rich Agricultural Products, School of Modern Industry for Selenium Science and Engineering, Wuhan Polytechnic University, Wuhan 430023, China; shuyi.li@whpu.edu.cn (S.L.); zhenzhouzhu@126.com (Z.Z.)

**Keywords:** yellowfin tuna pancreas, Alzheimer’s disease, AChE inhibitory peptides, moderate ultrasound-assisted enzymatic hydrolysis, peptide-enzyme interaction

## Abstract

Bioactive peptides represent a promising therapeutic approach for Alzheimer’s disease (AD) by maintaining cholinergic system homeostasis through the inhibition of acetylcholinesterase (AChE) activity. This study focused on extracting AChE inhibitory peptides from yellowfin tuna pancreas using moderate ultrasound-assisted enzymatic hydrolysis (MUE). Firstly, papain and MUE stood out from five enzymes and four enzymatic hydrolysis methods, respectively, by comparing the degree of hydrolysis and AChE inhibitory activity of different pancreatic protein hydrolysates. Subsequently, the optimal MUE conditions were obtained by single-factor, Plackett–Burman, and response surface methodologies. The pancreatic protein hydrolysate prepared under optimal MUE conditions was then purified by ultrafiltration followed by RP-HPLC, from which a novel AChE inhibitory peptide (LLDF) was identified by LC-MS/MS and virtual screening. LLDF effectively inhibited AChE activity by a competitive inhibition mechanism, with an IC_50_ of 18.44 ± 0.24 μM. Molecular docking and molecular dynamic simulation revealed that LLDF bound robustly to the active site of AChE via hydrogen bonds. These findings provided a theoretical basis for the valuable use of yellowfin tuna pancreas and introduced a new viewpoint on the potential therapeutic advantages of AChE inhibitory peptides for future AD treatment.

## 1. Introduction

Alzheimer’s disease (AD) is a chronic neurodegenerative disorder primarily characterized by cognitive and memory dysfunction [1]. The cholinergic hypothesis reveals that AD is closely associated with the loss of cholinergic neurons. Elevated activity of acetylcholinesterase (AChE) catalyzes the excessive breakdown of acetylcholine (ACh), leading to the disruption of cholinergic transmission and consequent damage to neurotransmission and memory function [2]. Therefore, AChE inhibitors for maintaining the brain ACh level have become a crucial strategy for preventing and treating AD [3]. Despite the effective effect of current clinical AChE inhibitors (tacrine, donepezil, rivastigmine, and galantamine) on regulating brain ACh level, they are all accompanied by side effects, such as nausea, vomiting, and even hepatotoxicity [4]. Bioactive peptides extracted from natural foods exerted an improvement effect on memory deficits with minimal toxicity and side effects [5,6,7]. Peptides derived from yellow field pea protein have been reported to exhibit good AChE inhibitory activity [8]. Anchovy peptides showed AChE inhibitory activity in vitro, protected PC12 cells against glutamate toxicity [9], and alleviated scopolamine-induced memory deficits in mice by up-regulating brain neurotransmitter levels, including ACh [10]. These studies indicated that food-derived bioactive peptides hold considerable potential as AChE inhibitors in the treatment of AD.

Although enzymatic hydrolysis is a common method for producing bioactive peptides, it still has limitations, including low yield and long processing time [11,12]. Ultrasound, a high-frequency mechanical wave, has been utilized as an assistant technique to enhance the enzymatic hydrolysis efficiency of bioactive peptides [13,14,15]. Compared with conventional enzymatic hydrolysis, ultrasonic-assisted enzymatic hydrolysis significantly improved the degree of hydrolysis (DH) and antioxidant capacity of potato protein hydrolysate [16]. The angiotensin-converting enzyme (ACE) inhibitory activity of rice protein hydrolysate obtained from ultrasound-assisted alkaline enzyme hydrolysis was significantly higher than that of hydrolysate obtained from conventional alkaline enzyme hydrolysis [17]. Ultrasonic assistance in enzymatic hydrolysis enhanced the enzymatic rate and promoted the conversion of proteins into target peptides by generating mechanical, cavitation, and thermal effects [16]. However, Hu et al. [18] found that long-term ultrasonic treatment in enzymatic hydrolysis of soy protein resulted in the aggregation of peptides and decreased the solubility of peptides. Wen et al. [19] and Wang et al. [20] also found that the DH and antioxidant capacity or ACE inhibitory activities of the ultrasound-assisted enzymatic protein hydrolysate of watermelon seed and oat initially increased and then decreased with increasing ultrasonic duration. These studies indicated that long-term ultrasound may result in peptide aggregation and a reduction in enzyme activity, ultimately leading to a decline in the yield and biological activity of peptides [21]. Thus, moderate ultrasound-assisted enzymatic hydrolysis initially to break protein structure and increase enzyme activity, followed by conventional enzymatic hydrolysis for the subsequent process, may be more efficient and energy-saving for preparing bioactive peptides. However, to our knowledge, there is limited research on the preparation of bioactive peptides using moderate ultrasonic-assisted enzymatic hydrolysis.

Marine animals are important sources of bioactive peptides [22,23,24]. Yellowfin tuna is a marine fish with high nutritional and economic value [25]. The processing of yellowfin tuna produces a large number of visceral by-products that are usually discarded, of which the pancreas accounts for about 0.82–1.18% of the whole fish [26]. Notably, yellowfin tuna pancreas contains 52.94% protein (dry weight) [26], and its protein hydrolysate exhibited antioxidant activity in vitro and protected against H_2_O_2_-induced oxidative damage in insulinoma cells dose-dependently [22]. The hypoglycemic and hypolipidemic activities of yellowfin tuna pancreatic protein hydrolysate have also been shown in diabetic rats and mice, respectively [24,27]. Although these previous studies have revealed that the pancreas of yellowfin tuna is a viable source for producing bioactive peptides, there are still gaps in research on AChE inhibitory peptides from yellowfin tuna pancreas. Therefore, this study aimed to prepare AChE inhibitory peptides from yellowfin tuna pancreas using moderate ultrasound-assisted enzymatic hydrolysis and further investigate the interaction between peptides and AChE. This study will promote the high-quality utilization of yellowfin tuna side products and provide important insights into the use of marine-derived AChE inhibitory peptides in Alzheimer’s disease.

## 2. Results and Discussion

### 2.1. Screening of Enzymes

**Figure 1:**
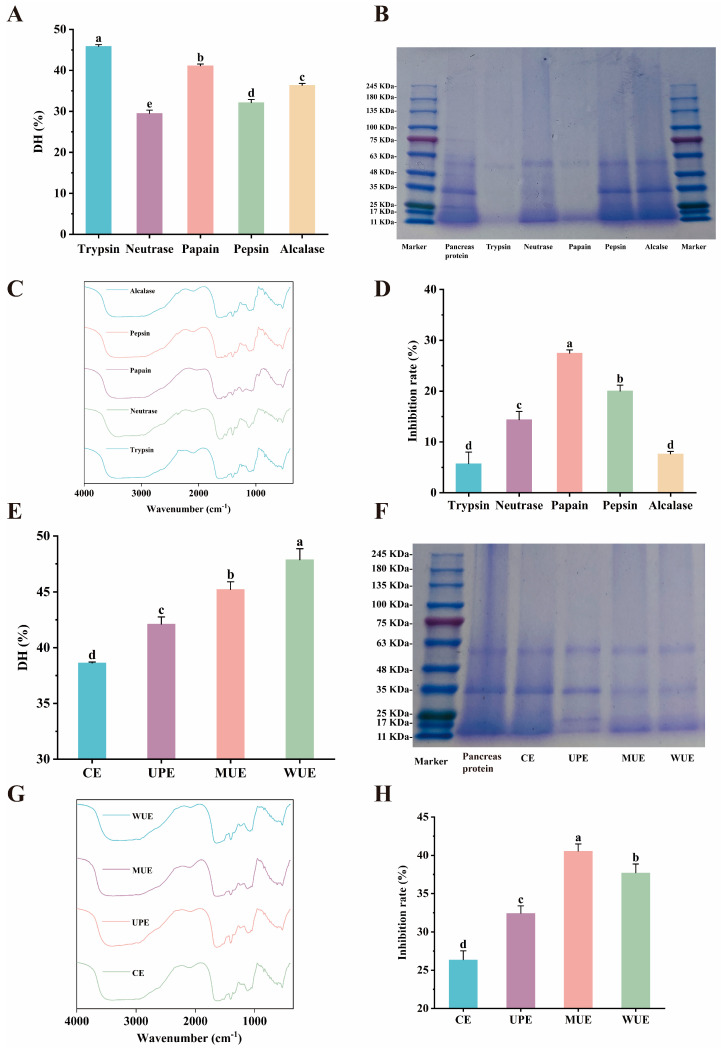
The effect of different enzymes and enzymatic hydrolysis methods on pancreatic protein hydrolysates. The DH (**A**), SDS-PAGE profile (**B**), FT-IR spectra (**C**), and AChE inhibitory activity (**D**) of pancreatic protein hydrolysates prepared by different enzymes; the DH (**E**), SDS-PAGE profile (**F**), FT-IR spectra (**G**), and AChE inhibitory activity (**H**) of pancreatic protein hydrolysates prepared by different enzymatic hydrolysis methods (CE = conventional enzymatic hydrolysis; UPE = ultrasonic pretreatment coupled with enzymatic hydrolysis; MUE = moderate ultrasonic-assisted enzymatic hydrolysis; WUE = whole process ultrasonic-assisted enzymatic hydrolysis). Means with different letters differ significantly at *p* < 0.05.

Enzymatic hydrolysis, as a method for preparing AChE inhibitory peptides, has the advantages of high specificity and simplicity of operation [28]. As shown in Figure 1A, trypsin-generated protein hydrolysate achieved the highest DH (45.85 ± 0.42%), followed by papain-generated protein hydrolysate with a DH of 41.11 ± 0.44% (*p* < 0.05).

The electrophoretic profile of pancreatic protein hydrolysates prepared by five different enzymes is presented in Figure 1B. The disappearance of the main proteins was particularly notable after enzymatic hydrolysis, especially in the pancreatic protein hydrolysates produced by trypsin and papain. Figure 1C showed the FT-IR spectra of five different pancreatic protein hydrolysates. The characteristic protein absorption peaks of the trypsin- and papain-generated protein hydrolysates were weaker than those of the other groups. This evidence supported the results of DH and FT-IR analysis, indicating that trypsin and papain were effective in hydrolyzing yellowfin tuna pancreas crude protein into smaller fragments. As illustrated in Figure 1D, the papain-generated protein hydrolysate demonstrated the most pronounced AChE inhibitory activity, exhibiting a range of 1.37 to 4.80 times higher than that observed in the other protein hydrolysates (*p* < 0.05). Although both trypsin- and papain-generated protein hydrolysates exhibited high DH, trypsin-generated protein hydrolysate showed the lowest inhibitory activity against AChE. This phenomenon may be attributed to the variations observed in particular cleavage sites. The hydrolysis of a single protein substrate by multiple enzymes can result in the production of peptides with disparate structures and biological activities [29]. Papain is regarded as a highly specific enzyme for the release of peptides containing terminal Lys and Arg [30], which is beneficial to inhibit AChE activity. Taking AChE inhibitory activity and DH into consideration, papain was selected for further research.

### 2.2. Enzymatic Hydrolysis Method Screening

As shown in Figure 1E, the DH of the UPE group was 1.09 times higher than that of the CE group (*p* < 0.05). The UPE group showed a lighter main protein band and a weaker characteristic protein absorption peak compared to the CE group (Figure 1F,G). Moreover, the AChE inhibitory activity of the UPE group was 1.30-fold higher than that of the CE group (*p* < 0.05, Figure 1H). These results revealed that ultrasound pretreatment contributed to the enzymatic hydrolysis of pancreatic crude protein, which aligned with the previous studies. Ultrasound pretreatment has been reported to promote the opening of the tight structure of the macromolecules [31]. Li et al. [32] found that ultrasonic pretreatment increased the DH of egg yolk powder by 106.28% compared to conventional enzymatic hydrolysis. Furthermore, ultrasonic pretreatment significantly increased the antioxidant activity of walnut protein isolate enzymatic hydrolysate [33].

The DH of the MUE group was 1.07-fold higher than that of the UPE group (*p* < 0.05, Figure 1E). The main protein band of pancreatic protein hydrolysate in the MUE group was lighter, and the characteristic absorption peak of the main protein was weaker compared to the UPE group (Figure 1F,G). The AChE inhibitory activity of the MUE group was significantly higher than that of the UPE group (*p* < 0.05, Figure 1H). These results indicated that the enzymatic hydrolysis effect of MUE on pancreas crude protein was superior to that of UPE. Rathod et al. [13] reported that ultrasound-assisted enzymatic hydrolysis can act directly on both the enzyme-substrate system and raw material, while ultrasonic pretreatment acts only on the raw material. In the MUE group, ultrasound assistance may allow both pancreas crude protein and papain to unfold more simultaneously, increasing the likelihood of protein binding to papain [34].

The DH of the MUE group (45.22 ± 0.69%) was 0.94-fold of that of the WUE group (47.87 ± 0.99%, *p* < 0.05, Figure 1E). The main protein bands of the MUE and WUE groups disappeared, and the characteristic peaks of their proteins were obviously weaker than those of the UPE and CE groups (Figure 1F,G). Notably, the MUE group exhibited considerably higher AChE inhibitory activity (40.54 ± 0.94%) compared to the WUE group (37.72 ± 1.15%) (*p* < 0.05, Figure 1H). The difference in ultrasonic duration between the MUE and WUE groups resulted in a lower DH but a higher AChE inhibitory activity in the MUE group than the WUE group. Previous studies reported that ultrasound-assisted enzymatic hydrolysis with appropriate time increased the likelihood of substrate binding to the enzyme [35], whereas long-term ultrasound-assisted enzymatic hydrolysis resulted in the aggregation of peptides and the decline of enzymatic activity [21]. The DH and ACE inhibitory activity of the ultrasound-assisted enzymatic hydrolysate of wheat gluten initially increased and then decreased with increasing ultrasonic duration [36]. Similar trends were also observed in the ultrasound-assisted enzymatic hydrolysis of watermelon seed and oat protein [19,20]. Additionally, the high temperature and pressure caused by the long-term ultrasound can break down H_2_O to -OH free radicals [37]. Long-term accumulation of free radicals results in glycosylation and further inactivation of enzymes [38]. Therefore, MUE was comprehensively selected as the method for enzymatic hydrolysis of yellowfin tuna pancreas crude protein.

### 2.3. Optimization of the MUE Process

#### 2.3.1. Single-Factor Experiment

(1) Ultrasonic power

**Figure 2:**
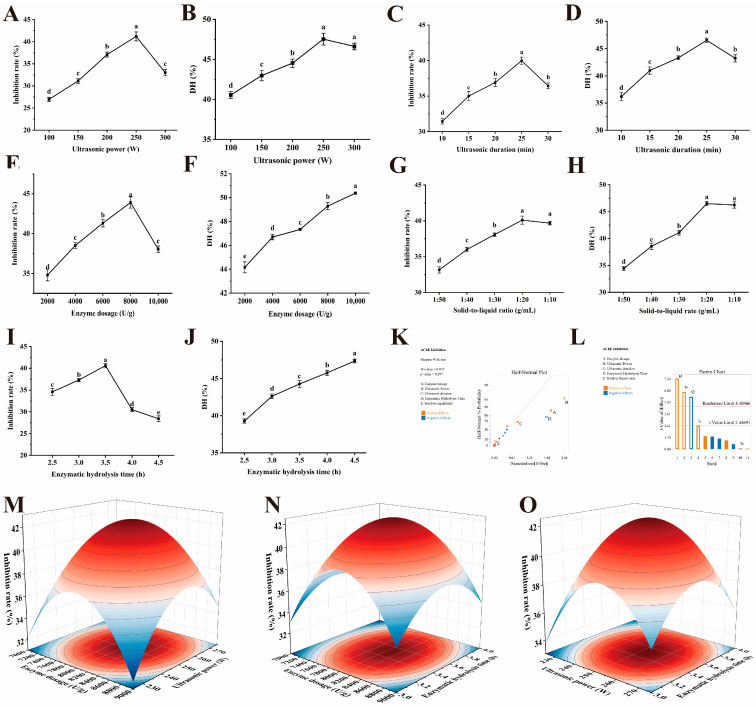
Optimization of MUE conditions for pancreatic protein hydrolysate. The effect of ultrasonic power (**A**), ultrasonic duration (**C**), enzyme dosage (**E**), solid-to-liquid ratio (**G**), and enzymatic hydrolysis time (**I**) on AChE inhibitory activity; the effect of ultrasonic power (**B**), ultrasonic duration (**D**), enzyme dosage (**F**), solid-to-liquid ratio (**H**), and enzymatic hydrolysis time (**J**) on DH; Standardized effect semi-normal probability plot (α = 0.05) (**K**); standardized effect Pareto plot (α = 0.05) (**L**); interaction effect of ultrasonic power and enzyme dosage on AChE inhibitory activity (**I**); interaction effect of ultrasonic power and enzymatic hydrolysis time on AChE inhibitory activity (**J**); interaction effect of ultrasonic power and enzyme dosage on AChE inhibitory activity (**M**); interaction effect of ultrasonic power and enzymatic hydrolysis time on AChE inhibitory activity (**N**); interaction effect of enzyme dosage and enzymatic hydrolysis time on AChE inhibitory activity (**O**). Means with different letters differ significantly at *p* < 0.05.

As shown in Figure 2A,B, as the ultrasonic power was increased from 100 W to 250 W, the AChE inhibitory activity of pancreatic protein hydrolysate increased from 26.98 ± 0.47% to 41.19 ± 1.01% (*p* < 0.05), and the DH increased from 40.55 ± 0.43% to 47.54 ± 0.74% (*p* < 0.05).

However, when the ultrasonic power was further increased to 300 W, the AChE inhibitory activity decreased to 33.02% (*p* < 0.05), with no significant change in DH (*p* > 0.05). Ultrasound treatment with appropriate power can improve the enzyme activity and loosen the protein structure, exposing more cleavage sites [39,40]. In contrast, excessive ultrasonic power generates a large amount of heat and energy, which can disrupt the conformation of the enzyme and reduce its activity [38]. Consequently, in this study, the AChE inhibitory activity and DH of pancreatic protein hydrolysate increased with the increasing ultrasonic power, while excessive ultrasonic power led to a decrease in DH of the pancreatic protein hydrolysate [41]. A similar trend was also observed in the study on preparing peanut and mung bean protein antioxidant peptides by ultrasound-assisted enzymatic hydrolysis [15]. Therefore, ultrasonic powers of 200 and 250 W were selected for the Plackett–Burman test.

(2) Ultrasonic duration

The ultrasonic duration significantly influenced AChE inhibitory activity and DH of pancreatic protein hydrolysate (*p* < 0.05, Figure 2C,D). When the ultrasonic duration increased from 10 to 25 min, there was a significant increase in AChE inhibitory activity (from 31.43 ± 0.36% to 39.98 ± 0.50%) and DH (from 36.18 ± 0.67% to 46.53 ± 0.40%) (*p* < 0.05). In contrast, when the ultrasonic duration was extended from 25 to 30 min, there was a significant reduction in both AChE inhibitory activity and DH (*p* < 0.05). Similarly, Wen et al. [19] reported that the DH and reducing power of watermelon seed peptides showed a trend of increasing first and then decreasing. Likewise, the DH and antioxidant activity of peanut protein hydrolysate rose first and then declined with the extended ultrasonic duration [41]. Long-term ultrasound may cause the aggregation of proteins and peptides, resulting in reduced enzyme activity and fewer sites for free radical reactions [38]. Thus, ultrasonic durations of 20 and 25 min were selected for the subsequent Plackett–Burman test.

(3) Enzyme dosage

As the enzyme dosage increased, DH progressively rose from 44.15 ± 0.46% to 50.37 ± 0.90%. The AChE inhibitory activity initially increased and then decreased with the increasing enzyme dosage (*p* < 0.05, Figure 2E,F). Specifically, as enzyme dosage increased from 2000 to 8000 U/g, AChE inhibitory activity rose from 34.81 ± 0.74% to 43.91 ± 0.70% (*p* < 0.05), peaking at 8000 U/g. At this dosage, AChE inhibitory activity of pancreatic protein hydrolysate was 1.26, 1.14, 1.06, and 1.15 times higher than that at dosages 2000, 4000, 6000, and 10,000 U/g, respectively (*p* < 0.05). Similarly, Hu et al. [42] reported that the antioxidant activity of *Gracilariopsis lemaneiformis* peptides increased first and then decreased with the increasing enzyme dosages. An appropriate papain dosage could effectively promote the enzymatic hydrolysis of pancreatic crude protein, resulting in an increase in DH and AChE inhibitory activities of pancreatic protein hydrolysate. In contrast, excessive enzyme dosage can lead to over-proteolysis, degrading the protein into oligopeptides and free amino acids with low or no AChE inhibitory activity [43], which led to an increase in DH but a decrease in AChE inhibitory activity. Consequently, enzyme dosages of 6000 and 8000 U/g were finally selected for further Plackett–Burman tests.

(4) Solid-to-liquid ratio

As the solid-to-liquid ratio increased from 1:50 to 1:20 mg/mL, there was a significant increase in AChE inhibitory activity (from 33.16 ± 0.44% to 40.10 ± 0.56%) and DH (from 34.43 ± 0.39% to 46.47 ± 0.35%, *p* < 0.05, Figure 2G,H). Subsequently, the AChE inhibitory activity and DH reached equilibrium at a solid-to-liquid ratio of 1:10 mg/mL (*p* > 0.05). At excessively high solid-to-liquid ratios, the pancreas crude protein may dissolve insufficiently and thus contact inadequately with papain. This inadequate contact slowed the reaction and limited enzymatic efficiency improvements [44]. Therefore, to optimize raw material usage and enhance preparation efficiency, the solid-to-liquid ratios of 1:30 and 1:20 mg/mL were selected for further Plackett–Burman test.

(5) Enzymatic hydrolysis time

The enzymatic hydrolysis time had a significant impact on AChE inhibitory activity and DH of pancreatic protein hydrolysate (*p* < 0.05, Figure 2I,J). As enzymatic hydrolysis time increased, DH rose from 39.28 ± 0.29% to 49.36 ± 0.25% (*p* < 0.05). As the enzymatic hydrolysis time increased from 2.5 h to 3.5 h, AChE inhibitory activity increased from 34.59 ± 0.83% to 40.61 ± 0.51% (*p* < 0.05), peaking at 3.5 h. The AChE inhibitory activity obtained from the enzymatic hydrolysis time of 3.5 h was 1.17, 1.08, 1.33, and 1.43 times higher than that obtained from 2.5, 3.0, 4.0, and 4.5 h, respectively (*p* < 0.05). Prolonged enzymatic hydrolysis time allowed the gradual breakdown of pancreatic crude protein into peptides with AChE inhibitory activity. However, beyond 3.5 h, peptides with AChE inhibitory activity were further degraded into smaller inactive fragments [45], resulting in the decline of AChE inhibitory activity. Considering these factors, enzymatic hydrolysis times of 3.0 and 4.0 h were selected for further Plackett–Burman tests.

#### 2.3.2. Plackett–Burman Test

In this study, a two-level Plackett–Burman factorial design with 12 runs was introduced to screen out in an unbiased manner the important variables that may have a marked effect on AChE inhibitory activity. The screening result demonstrated that the AChE inhibitory activity varied widely, ranging from 34.22 to 41.49% in 12 trials (Table S1). Significant effects were identified by the Lenth method [46]. Figure 2K,L illustrated the standardized Pareto charts (*p* < 0.05) and a semi-normal probability effect plot of the main effects. As shown in Table 1, three variables, including enzyme dosage (*p* < 0.01), ultrasonic power (*p* < 0.0001), and enzymatic hydrolysis time (*p* < 0.01), were significant factors for AChE inhibitory activity. Among them, enzyme dosage and ultrasonic power were significant factors with positive effects, while enzymatic hydrolysis time had a negative effect. Ultrasonic duration (*p* > 0.05) and solid-to-liquid ratio (*p* > 0.05) were non-significant factors for AChE inhibitory activity. The magnitude and significance of these effects were indicated by ultrasonic power, enzyme dosage, and enzymatic hydrolysis time exceeding the critical t-value in the Pareto chart [47]. Consequently, the enzyme dosage, ultrasonic power, and enzymatic hydrolysis time were chosen for further RSM optimization, and the remaining variables were maintained at low levels (ultrasonic duration of 20 min and solid-to-liquid ratio of 1:30 mg/mL).

#### 2.3.3. RSM

As shown in Table 2, the AChE inhibitory activity of pancreatic protein hydrolysate varied from 31.31% to 42.59% across these trials. Notably, in five central trials, the AChE inhibitory activity remained stable at approximately 42.3%.

A quadratic polynomial regression model was applied to the AChE inhibitory activity, producing regression equations for AChE inhibitory activity relative to enzyme dosage (A), ultrasonic power (B), and enzymatic hydrolysis time (C):

(1) $$\begin{array}{c} Y=-868.7345+0.082221A+3.27415B+95.126C+0.000031AB+0.000255AC-0.0222BC-0.00000563950A^{2}\\ -0.006823B^{2}-13.028C^{2} \end{array}$$

Variance analysis of the regression equation was detailed in Table 3. The regression equation aligned closely with the experimental data (*p* < 0.001). Consequently, the regression equation could be utilized to analyze and predict the actual values of AChE inhibitory activity. The adjusted coefficient of determination (R_Adj_^2^) was 0.9787, indicating that approximately 97.87% of the variation in AChE inhibitory activity was attributable to the three examined factors, with 2.13% unexplained by the model. In the case of the actual and predicted values, the correlation coefficient (R^2^) was 0.9907, showing a strong correlation. The lack of fit was 0.0657, suggesting the consistency of experimental results with the response surface model. Additionally, the coefficient of variability (CV) of this model was 1.73%, further indicating a good fit for the regression equation. The design of level intervals for the studied factors was reasonable, as evidenced by an adequate precision value of 22.63.

The significance of the regression coefficients in the quadratic model is presented in Table 3. The linear coefficients for A and B on AChE inhibitory activity were found to be statistically significant (*p* < 0.05), as well as the interaction between AB and AChE inhibitory activity (*p* < 0.05). The quadratic effects of A^2^, B^2^, and C^2^ on AChE inhibitory activity were highly significant (*p* < 0.0001, Figure 2M–O). These results suggested that the relationship between these factors and AChE inhibitory activity was complex and non-linear. Both quadratic terms and interactions exerted a considerable impact on the dependent variable. A comparison of the regression equation’s coefficients revealed the following order of influence on AChE inhibitory activity of pancreatic protein hydrolysate: B (ultrasonic power) > A (enzyme dosage) > C (enzymatic hydrolysis time).

Optimal conditions for the MUE preparation of pancreatic protein hydrolysate were identified as follows: ultrasonic power of 253.35 W, enzyme dosage of 8053.12 U/g, and enzymatic hydrolysis time of 3.52 h. Under these optimized conditions, the predicted value of AChE inhibitory activity could reach 42.51%. To confirm the feasibility of the RSM, verification tests were conducted. Based on practical operational factors and convenience, the process parameters have been slightly adjusted as follows: ultrasonic power of 253 W, enzyme dosage of 8053 U/g, and enzymatic hydrolysis time of 3.52 h. Under these conditions, the AChE inhibitory activity was measured to be 42.75 ± 0.78%. The relative error between the predicted and actual AChE inhibitory activity was less than 2%, indicating an excellent fit between the predicted and experimental values and confirming the model’s predictive suitability.

### 2.4. Purification

**Figure 3:**
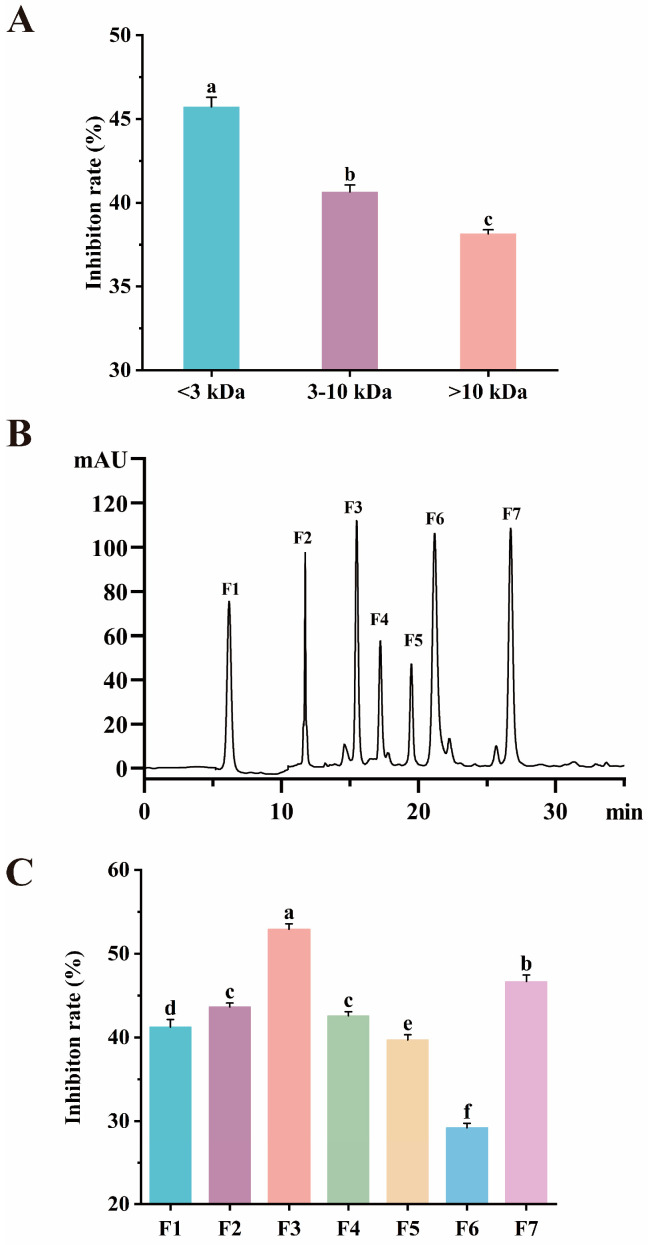
Purification and identification of AChE inhibitory peptide. AChE inhibitory activity of different ultrafiltration fractions (**A**); RP-HPLC chromatogram (**B**); AChE inhibitory activity of each RP-HPLC fraction (**C**). Different letters indicate significant differences. Means with different letters differ significantly at *p* < 0.05.

Purification is critical for isolating peptides with strong AChE inhibitory activity [9,48,49]. In this study, ultrafiltration and RP-HPLC were employed to purify the pancreatic protein hydrolysate. Specifically, the AChE inhibitory activity of the ultrafiltration fraction with MW < 3 kDa was 1.13 and 1.20 times higher than that of the fractions with MW = 3–10 kDa and MW > 10 kDa, respectively (*p* < 0.05, Figure 3A). RP-HPLC further separated the ultrafiltration fraction with MW < 3 kDa into seven fractions (Figure 3B). Among the fractions, fraction 3 (F3) exhibited the highest AChE inhibitory activity of 52.93 ± 0.65% (*p* < 0.05, Figure 3C), which was 1.24-fold that of the unpurified pancreatic protein hydrolysate (*p* < 0.05). After anchovy protein hydrolysate was purified using RP-HPLC, its AChE inhibitory activity was significantly increased from 12.06 ± 0.36% to 23.30 ± 0.97% [9]. Similarly, Asen et al. [49] demonstrated that the AChE inhibitory activity of yellow field pea hydrolysate significantly increased after being purified by RP-HPLC. These findings highlighted the importance of purification in obtaining peptides with good biological activity.

### 2.5. Identification and Screening of AChE Inhibitory Peptides

**Figure 4:**
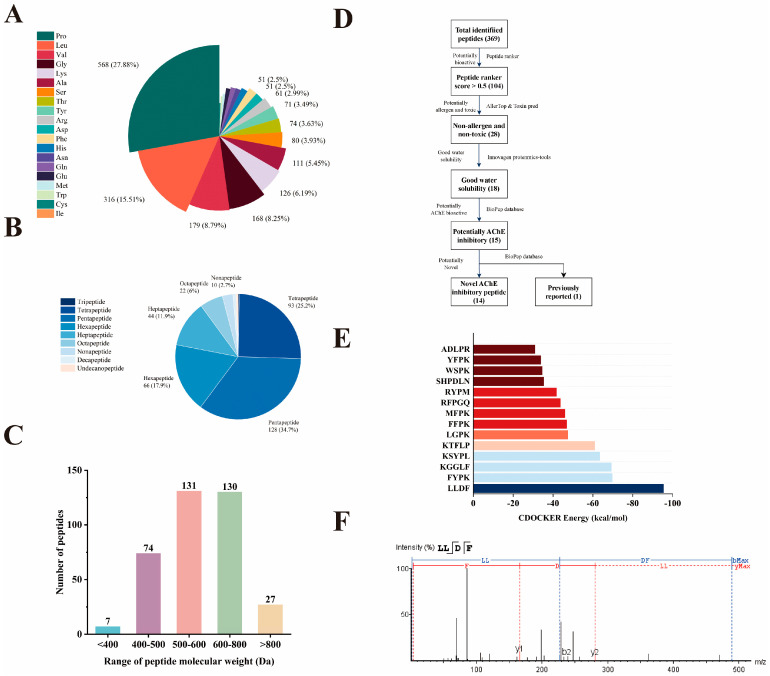
Virtual screening of novel AChE inhibitory peptides. Virtual screening procedure (**A**); amino acid composition of the detected peptides (**B**); MW distribution of the detected peptides (**C**); proportions of peptides with varying lengths (**D**); molecular docking CDOCKER energy (**E**); and MS/MS spectrum of LLDF (**F**).

A total of 369 peptide sequences were identified from F3 using LC-MS/MS. As shown in Figure 4A, most of these peptides contained hydrophobic amino acid residues (65.34%) and basic amino acid residues (11.14%). Most of the reported AChE inhibitory peptides identified from hemp seeds [50], soybeans [49], and walnuts [51] contained these two types of amino acid residues. In the peptides identified from F3, the aromatic amino acid residues (Trp, Tyr, and Phe) accounted for 6.77% of the total amino acids, which have been reported to exhibit significant AChE affinity and potential neuroprotective effects [52]. Moreover, 6.68% of the peptides contained Phe/Ile/Leu/Trp residues at the N-terminus. The structure-activity relationship studies have shown that this feature is closely related to AChE inhibition [51]. In addition, most peptides were composed of 4–5 amino acid residues with MW between 400–600 Da (Figure 4B,C). Previous studies reported that the AChE inhibitory peptides, such as PAYCS [9], SQSQ [49], KLPGF [53], and ARSR [54], from anchovy, yellow field pea, albumin, and pea protein were composed of 4–5 amino acid residues with MW between 400–600 Da, respectively.

Virtual screening was used to screen for potential AChE inhibitory peptides from F3 (Figure 4D). Firstly, the biological activity of the 369 peptide sequences was predicted by Peptide Ranker, with 104 peptides scoring above 0.5, indicating a high probability of biological activity [55]. Considering the importance of safety in the application of bioactive peptides [56], only 28 peptides predicted to be non-allergenic and non-toxic were retained. Furthermore, a significant correlation exists between peptide solubility and its bioavailability [57]. Among the 28 non-allergenic and non-toxic peptides, only 15 peptides exhibited good water solubility. Among them, KLPGF has been identified in albumin and demonstrated to possess strong AChE inhibitory activity [53]; thus, the remaining 14 peptides were identified as novel potential AChE inhibitory peptides.

Molecular docking was employed to predict the binding affinity of the chosen 14 peptides to AChE. As illustrated in Figure 4E, all peptides exhibited the ability to bind the active site of AChE, with low CDOCKER energy values ranging from −30.86 to −95.39 kcal/mol. Notably, among these 14 peptides, LLDF had the lowest CDOCKER energy value of −95.39 kcal/mol, which was considerably lower than that of previously reported AChE inhibitory peptides, such as WIR from fish roe (−76.51 kcal/mol) [58] and KLPGF from albumin (−20.52 kcal/mol) [53]. Consequently, LLDF was selected for further investigation of its AChE inhibitory effects, and its MS/MS spectrum is shown in Figure 4F.

### 2.6. Synthesis of LLDF and Confirmation of AChE Inhibitory Activity

#### 2.6.1. Inhibition Kinetics of LLDF on AChE

**Figure 5:**
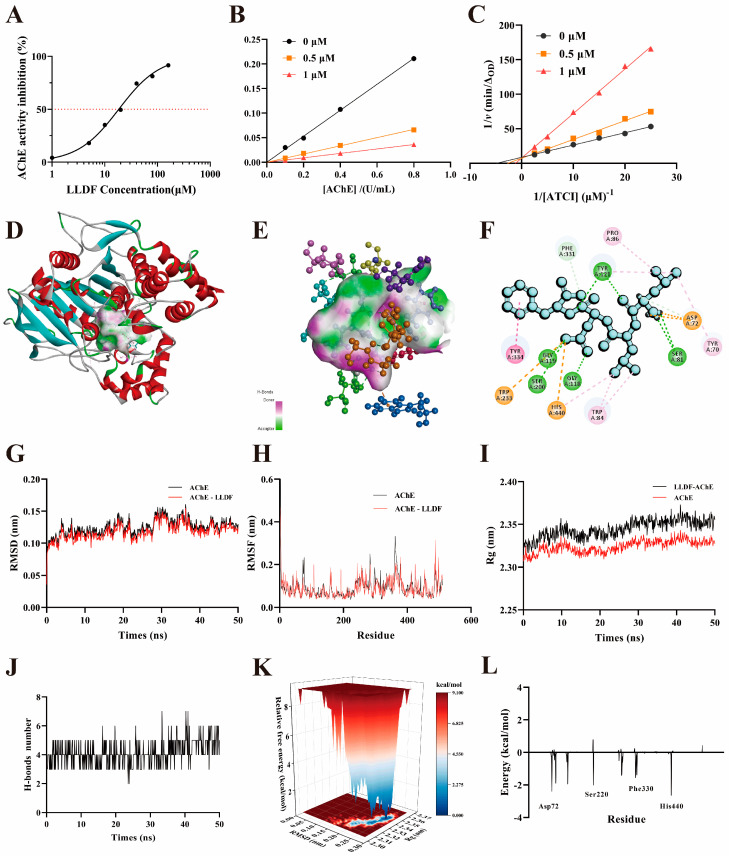
The interaction between LLDF and AChE. Validation of the AChE inhibitory activity of LLDF (**A**); the type of LLDF inhibition by AChE (**B**); lineweaver-Burk plots for AChE inhibition by LLDF (**C**); interactions between LLDF and AChE (**D**); binding geometry of LLDF in the active site of AChE (**E**); three-dimensional binding mode of LLDF with AChE (green lines represent hydrogen bonds; blue lines represent carbon-hydrogen bonds; yellow lines represent pi-alkyl interactions; pink lines represent pi-pi stacking) (**F**); the RMSD (**G**), RMSF (**H**), Rg (**I**), and number of hydrogen bonds (**J**) of the LLDF-AChE complex during 50 ns molecular dynamics simulation; free energy landscape (**K**); contribution of AChE amino acid residues to the total binding energy in the LLDF-AChE complex (**L**).

As depicted in Figure 5A, LLDF exerted a dose-dependent inhibitory activity against AChE, with an IC_50_ value of 18.44 ± 0.24 μM. The IC_50_ values of priorly identified peptides WIR, DNRMLRTTRY, and KLSPSLGPVSKGKLLAGQR from fish roe, the virtual design approach, and the skin secretions of an Argentinian frog were 43.32, 51.20, and 89.80 μM [58,59,60], respectively. LLDF showed a more potent AChE inhibitory activity than these previously recorded peptides. There is probably a relationship between the amino acid composition of the different peptides and their AChE inhibitory activities. A further investigation of the structure-activity relationship between peptides and AChE inhibition is needed.

The initial rate of the AChE-mediated reaction was analyzed in relation to AChE concentration at different LLDF concentrations to evaluate the reversibility of LLDF inhibition. As shown in Figure 5B, as the concentration of LLDF increased, the slope of the curve demonstrated a decline, which indicated a reversible inhibition of AChE by LLDF. The findings indicated that LLDF might reversibly attach to AChE or the AChE-substrate complex via a non-covalent mechanism. This could result in a reduction in the catalytic activity of AChE and, subsequently, the rate of the reaction process [61].

Competitive, non-competitive, and mixed inhibitory patterns are predominant in inhibitors [61]. To ascertain the inhibitory pattern of LLDF on AChE, double reciprocal plots were constructed using the Lineweaver-Burk method (Figure 5C). It could be found that the slopes of the straight lines increased with the increasing concentrations of LLDF, and the three straight lines intersected each other on the *y*-axis. The V_max_ (obtained from vertical axis intercept values) was not changed by the presence of LLDF, but K_m_ (obtained from horizontal axis intercept values) was increased with the increase of LLDF concentrations. These confirmed that LLDF inhibited AChE by typical competitive inhibition mode and competitively bound to the active site of AChE along with the substrate ATCI. The similarity of the binding sites of LLDF and ATCI to AChE’s active site may explain this competitive behavior. A novel peptide derived from spider venom was also a competitive-type inhibitor of AChE [62].

#### 2.6.2. Molecular Docking

Molecular docking has proven to be a useful method for the investigation of intermolecular interactions between the peptide and enzyme [63]. In this study, the interaction between LLDF and AChE was investigated through the use of molecular docking. Figure 5D showed that LLDF was capable of embedding into the active site of AChE, effectively obstructing the entry of substrate ATCI. Furthermore, LLDF established hydrogen bonds with residues including Ser81, Tyr121, Gly118, Gly119, and Ser200 of AChE, formed a carbon-hydrogen bond with Phe331, and interacted with amino acid residues, such as Tyr70, Pro86, Trp84, and His440, to establish stable pi-alkyl interactions (Figure 5E,F). In addition, it also formed a pi-pi binding with Tyr334. The SAA from *Salvia miltiorrhiza* also mainly binds to AChE through several same amino acid residues, including Tyr70, Tyr121, Tyr334, Phe330, and His440 [64]. These findings suggested that LLDF could be a promising novel AChE inhibitor and highlighted a correlation between the mode of inhibition analysis and computational data predictions.

#### 2.6.3. Molecular Dynamic Simulations

Compared to molecular docking, molecular dynamics simulations could reflect the deeper interaction mechanisms between the ligand and receptor at the atomic level [65]. RMSD was commonly employed to assess structural changes and dynamic stability in ligand-receptor complexes [66]. The lower the RMSD value, the more stable the molecular conformation of the complex. Figure 5G showed the time-dependent RMSD profile of the LLDF-AChE complex during the 50 ns simulation. The average RMSD value of the LLDF-AChE complex was 0.107 ± 0.007 nm, which was lower than the RMSD value of native AChE (0.114 ± 0.011 nm), indicating that LLDF-AChE was relatively stable. This finding indicated that the entry of LLDF into AChE not only caused no significant fluctuations but also reduced fluctuations within AChE. RMSF values represented the average amplitude of movement of individual amino acid residues over time. Lower RMSF values indicated less vigorous movement under simulated conditions [67]. The RMSF value (0.077 ± 0.029 nm) of the LLDF-AChE complex was consistently below 0.5 nm during the 50 ns simulation, slightly lower than native AChE (0.080 ± 0.032 nm, Figure 5H). Rg reflected the compactness of the ligand-receptor binding; a smaller Rg indicated a more stable conformation [68]. As shown in Figure 5I, the average Rg of AChE was 2.319 nm, while the LLDF-AChE complex exhibited a slight increase to 2.336 nm, indicating that structural stability was maintained after binding. Figure 5J showed that the number of hydrogen bonds formed between LLDF and AChE remained relatively stable during the simulation, supporting the stability of the LLDF-AChE complex. Further validation was provided by the 2D free energy landscape (Figure 5K), with only a single stable energy well indicating the stable conformation of the LLDF-AChE complex under the set MD conditions [69].

The binding free energy of LLDF to AChE was calculated using the MM/PBSA method [70]. As shown in Table S2, the total free energy (ΔG_total_) was −18.1 kcal/mol, indicating a stable LLDF-AChE complex. The MM value (ΔG_vdw_ + ΔG_ele_) was 49.214 kcal/mol, indicating that electrostatic and van der Waals forces played crucial roles in the combination between LLDF and AChE. The contributions of individual amino acid residues to the binding energy were also assessed. As shown in Figure 5L, residues with large binding energy contributions, including His440 (−2.638 kcal/mol), Asp72 (−2.386 kcal/mol), Ser200 (−2.005 kcal/mol), Tyr121 (−1.939 kcal/mol), Trp84 (−1.882 kcal/mol), Phe330 (−1.551 kcal/mol), Ile287 (−1.405 kcal/mol), and Tyr334 (−1.093 kcal/mol), significantly contributed to maintaining the stability of the interaction between LLDF and AchE. Hence, the results of molecular dynamics simulations provided additional evidence based on molecular docking and suggested that the LLDF bound stably to AChE through a strong and stable interaction during molecular simulations.

## 3. Materials and Methods

### 3.1. Materials and Chemicals

Yellowfin tuna pancreas was purchased from Hainan Orca Sea Fishing Services Co., Ltd. (Hainan, China) and preserved at −80 °C. Papain, alkaline protease, neutral protease, trypsin, pepsin, sodium tetraborate, sodium dodecyl sulfate (SDS), ortho phthalaldehyde (OPA), serine, acetylthiocholine iodide (ATCI), trichloroacetic acid, 5,5′-dithiobis-(2-nitrobenzoic acid) (DTNB), and acetonitrile were provided by Shanghai Yuanye Biotechnology Co., Ltd. (Shanghai, China). Protein loading buffers and markers were purchased from Solarbio (Beijing, China). Omni-PAGE™ Hepes-Tris gel was supplied by Nanjing Aipusaisi Biotechnology Co., Ltd. (Shanghai, China).

### 3.2. Extraction of Pancreatic Crude Protein

Yellowfin tuna pancreas was thawed, rinsed under running water, homogenized, and then freeze-dried. The freeze-dried pancreas powder was mixed with isopropanol (1:15, *w*/*v*) and stirred at 25 °C for 6 h, replacing the isopropanol every 3 h. The mixture was centrifuged at 5000 rpm/min at 4 °C for 15 min, the precipitate was lyophilized, and then pancreas crude protein was obtained.

### 3.3. Effects of Different Enzymes on Pancreatic Protein Hydrolysates

Five enzymes (trypsin, neutral protease, papain, pepsin, and alkaline protease) were chosen for enzymatic hydrolysis of pancreatic crude protein according to the method of Yu et al. [48]. Briefly, pancreas crude protein was mixed with deionized water at a ratio of 1:25 (*w*/*v*), and enzymes (8000 U/g) were added [71]. The manufacturer recommends the following optimal conditions for enzymatic hydrolysis: trypsin, pH 8.1, temperature 37 °C; neutral enzyme, pH 7.0, temperature 50 °C; papain, pH 6.5, temperature 55 °C; pepsin, pH 3.0, temperature 37 °C; alkaline enzyme, pH 9, temperature 50 °C. Enzymatic hydrolysis was performed under constant agitation for 3 h, while pH was maintained within an optimal range for each enzyme by adding 1 M NaOH or HCl. Finally, the mixture was heated in a 95 °C water bath for 15 min to terminate the enzymatic hydrolysis reaction, and the supernatant was collected by centrifugation at 4 °C and 8000 rpm/min for 20 min to obtain pancreatic protein hydrolysate. The optimal enzyme was selected for subsequent experiments based on the AChE inhibitory activity and DH of pancreatic protein hydrolysate.

### 3.4. Effects of Different Enzymatic Hydrolysis Method on Pancreatic Protein Hydrolysates

To compare the effect of different enzymatic hydrolysis methods, pancreas crude protein was mixed with deionized water at a ratio of 1:25 (*w*/*v*) and enzymatically hydrolyzed using the following four methods. In the end, the mixture was placed in a 95 °C water bath for 15 min to terminate the enzymatic hydrolysis reaction and centrifuged at 8000 rpm/min at 4 °C for 20 min to obtain pancreatic protein hydrolysate. The optimal enzymatic hydrolysis method was selected for subsequent experiments based on the AChE inhibitory activity and DH of pancreatic protein hydrolysate.

(1) Conventional enzymatic hydrolysis (CE)

The pancreatic protein solution was stirred at 25 °C for 20 min, then papain was added at a dosage of 8000 U/g, and enzymatic hydrolysis was conducted at pH 6.5 and 55 °C for 180 min.

(2) Ultrasonic pretreatment coupled with enzymatic hydrolysis (UPE):

The pancreatic protein solution was ultrasonically pre-treated for 20 min at 25 °C using an ultrasonic cell crusher (SCIENTZ-IID, SCIENTZ, Ningbo, China). The ultrasonic conditions were set at an output power of 200 W, a temperature of 55 °C, a frequency of 25 kHz, and a pulse interval ratio of 0.5 s/0.5 s. Subsequently, papain was added at a dosage of 8000 U/g, and enzymatic hydrolysis was carried out at pH 6.5 and 55 °C for 180 min.

(3) Moderate ultrasonic-assisted enzymatic hydrolysis (MUE)

The pancreatic protein solution was stirred at 25 °C for 20 min, and then papain was added at a dosage of 8000 U/g. Subsequently, the mixture was subjected to ultrasonic-assisted enzymatic hydrolysis using an ultrasonic cell crusher for 20 min at pH 6.5. The ultrasonic conditions were set at an output power of 200 W, a temperature of 55 °C, a frequency of 25 kHz, and a pulse interval ratio of 0.5 s/0.5 s. Finally, the mixture underwent conventional enzymatic hydrolysis at 55 °C for an additional 160 min.

(4) Whole process ultrasonic-assisted enzymatic hydrolysis (WUE):

The pancreatic protein solution was stirred at 25 °C for 20 min and then papain was added at a dosage of 8000 U/g, and enzymatic hydrolysis was conducted with continuous ultrasonic assistance using an ultrasonic cell crusher at pH 6.5 and 55 °C for 180 min. The ultrasonic conditions were set at an output power of 200 W, a temperature of 55 °C, a frequency of 25 kHz, and a pulse interval ratio of 0.5 s/0.5 s.

### 3.5. Sodium Dodecyl Sulfate-Polyacrylamide Gel Electrophoresis (SDS-PAGE)

The effect of different enzymes and enzymatic hydrolysis methods on pancreas crude protein was observed by SDS-PAGE gel electrophoresis, with pancreas crude protein as a control. Each sample was diluted to a concentration of 30 mg/mL, mixed with the 5 × protein loading buffer at a ratio of 4:1 (*v*/*v*), and then boiled for 10 min to denature sufficiently. The samples were analyzed by SDS-PAGE using a 4–20% precast polyacrylamide gel, and electrophoresis was performed at 120 V for 40 min. Electrophoresis was stopped when the dye reached 1 cm above the bottom of the glass plate. The gel was removed, stained with Coomassie Brilliant Blue, and stained with destaining solution until the bands were clear.

### 3.6. Determination of DH

The DH was determined by the OPA method described by Liang et al. [72]. Briefly, the pancreatic protein hydrolysate (400 μL) was added into freshly prepared OPA solution (3 mL) and mixed for 5 s. After the mixture was incubated at 25 °C for 2 min, the absorbance of the mixture was measured at 340 nm using a UV spectrometer (UV-5100, Metash Instrument Co., Ltd., Shanghai, China). Serine was used as a standard. All samples were determined in triplicate.

### 3.7. Fourier Transform Infrared Spectroscopy (FT-IR)

The FT-IR spectra of pancreatic protein hydrolysate were determined by the method of Wu et al. [73] with appropriate modifications. The freeze-dried sample and KBr were ground at a ratio of 1:100 (*w*/*w*) and then pressed into tablets. The tablets were placed in an FT-IR (PerkinElmer Frontier, PerkinElmer, Inc., Hopkinton, MA, USA) for infrared spectroscopy with 32 scans and a resolution of 4 cm^−1^.

### 3.8. AChE Inhibitory Activity Assay

The AChE inhibitory activity of the sample was measured as described by Moreira et al. [74]. After phosphate buffer (90 μL), deionized water (45 μL), AChE (10 μL), and 10 μL of sample or phosphate buffer (control) were sequentially added into a 96-well plate, the mixture was incubated at 25 °C for 10 min. Subsequently, DTNB (20 μL) and ATCI (20 μL) were added to the mixture in the dark. The absorbance was measured at 412 nm using a BioTek Gen5^TM^ microplate reader (BioTek Gen5^TM^, Agilent, Santa Clara, CA, USA) every minute for 4 min. The AChE inhibitory activity was expressed as the percentage of activity relative to the control (100%).

### 3.9. MUE Process Optimization

#### 3.9.1. Single-Factor Experiment and Plackett–Burman Test

The effects of different parameters (ultrasonic power, ultrasonic duration, solid-to-liquid ratio, enzyme dosage, and enzymatic hydrolysis time) on the AChE inhibitory activity and DH were investigated to optimize the process of MUE by single-factor tests. Subsequently, the effect of these parameters on the AChE inhibitory activity was further investigated by the Plackett–Burman test.

#### 3.9.2. Response Surface Methodology (RSM)

Based on the results of the single-factor and Plackett–Burman tests, three significant factors influencing the AChE inhibitory activity most (enzyme dosage (A), ultrasonic power (B), and enzymatic hydrolysis time (C)) were further optimized using RSM, with three levels assigned to each factor.

### 3.10. Separation and Purification

#### 3.10.1. Ultrafiltration

The pancreatic protein hydrolysate was subjected to ultrafiltration, resulting in the isolation of three fractions. These fractionated samples were separated based on their molecular weight (MW) characteristics, with cut-offs of 10 kDa and 3 kDa employed. The three fractions (>10 kDa, 3–10 kDa, and <3 kDa) were then freeze-dried, and their AChE inhibitory activities were determined at a concentration of 10 mg/mL.

#### 3.10.2. RP-HPLC

The freeze-dried fraction (<3 kDa) was redissolved in deionized water. The separation was conducted on an RP-HPLC system (Agilent 1260, Agilent Technologies, CA, USA) equipped with a C18 column (20 × 250 mm, 10 μm, Agilent 1260, Agilent Technologies, CA, USA), with elution A (deionized water) and mobile elution B (methanol) in the following gradient procedure: 0–10 min, 5–10% B; 10–35 min, 10–15% B. The flow rate was set at 1 mL/min. After the fractions were collected and freeze-dried, their AChE inhibitory activities were measured at a concentration of 10 mg/mL.

### 3.11. Identification and Screening of Potential AChE Inhibitory Peptides

The peptide sequence of the RP-HPLC purified fraction displaying the greatest AChE inhibitory activity was identified through the use of LC-MS/MS, in accordance with the methodology described by Yu et al. [48]. The Supplementary Information provided a detailed account of the identification process. The biological activities of the identified peptides were predicted using Peptide Ranker (http://distilldeep.ucd.ie/PeptideRanker/ (accessed on 14 March 2024)). The potential toxicity and allergenicity of the peptides were evaluated using the ToxinPred tool (https://webs.iiitd.edu.in/raghava/toxinpred/design.php (accessed on 16 March 2024)) and Allercatpro (https://allercatpro.bii.a-star.edu.sg/ (accessed on 16 March 2024)), respectively. Additionally, the solubility of the peptides was predicted using an online bioactivity tool (https://web.expasy.org/protparam/ (accessed on 17 March 2024)). Molecular docking was performed using the CDOCKER protocol in Discovery Studio 2019, and energy minimization was carried out with the CHARMM force field. The docking process targeted the AChE active site (coordinates: x: 2.855803, y: 64.576958, z: 67.968147). The peptide-AChE complex with the lowest CDOCKER energy was selected for further analysis.

### 3.12. The Interaction Between LLDF and AChE

#### 3.12.1. Measurement of Half Maximal Inhibitory Concentration (IC_50_)

The novel AChE inhibitory peptide, LLDF, was synthesized by Jietai Biotechnology (Nanjing, China), and HPLC confirmed the purity of the peptide to be greater than 95%. The inhibitory activity of LLDF on AChE was evaluated at concentrations of 1, 5, 10, 20, 40, 80, and 160 µM, employing the methodology outlined in Section 3.8. Prism software 9.5.0 was used to calculate the IC_50_ of LLDF.

#### 3.12.2. Kinetics of AChE Inhibition

The objective of this study is to ascertain the reversibility of LLDF’s inhibitory effect on AChE, LLDF solutions (0, 0.5, and 1 µM) were mixed with AChE solutions (0 to 0.8 U/mL) and incubated at 37 °C for 15 min while maintaining a constant substrate ATCI concentration of 10 µM. The absorbance was determined at 412 nm in accordance with the methodology delineated in Section 3.8.

To ascertain the inhibitory pattern of LLDF on AChE, the Lineweaver-Burk equation was employed. As described in Section 3.8, the effect of LLDF (0, 0.5, and 1 µM) on AChE inhibitory activity was assayed with varying concentrations of ATCI (0.04 to 4 µM) to calculate the K_m_ and V_max_ values.

#### 3.12.3. Molecular Docking of LLDF and AChE

The crystal structure of AChE (PDB ID: 1EVE) was downloaded from the Protein Data Bank (PDB) and prepared by removing water molecules and adding hydrogen atoms. The structure of LLDF was drawn using Discovery Studio 2019 software, and molecular docking was performed using the CDOCKER protocol, followed by energy minimization with the CHARMM force field. The coordinates for the AChE active site used in the docking process were x: 2.855803, y: 64.576958, z: 67.968147.

#### 3.12.4. Molecular Dynamic Simulations of LLDF and AChE

Simulated molecular dynamics of the LLDF-AChE complex were performed using GROMACS 2018.4, with the Amber force field applied to the ligands and the SPC force field applied to the water molecules. Na^+^ and Cl^−^ ions were introduced to neutralize the system after it had been solvated with a water model. Position restraints were applied before the steepest descent and conjugate gradient algorithms were used to minimize energy. As a pre-equilibration step, NVT and NPT equilibration simulations were performed. In the next step, a molecular dynamics simulation of production was performed for 50 ns. The stability of the complex was analyzed by calculating the root mean square deviation (RMSD), radius of gyration (Rg), root mean square fluctuation (RMSF), and the number of hydrogen bonds. Free energy landscapes were constructed using principal component analysis (PCA) based on Rg and RMSD, and binding free energy was calculated using computational tools. Based on trajectories of 0 to 50 ns, conformation samples were taken every 100 ps, resulting in a total of 500 conformations.

### 3.13. Statistical Analysis

Data are expressed as mean ± standard deviation. Statistical analysis was performed using SPSS 27.0 statistical software. Single-factor ANOVA was used for comparisons among multiple groups, and Duncan’s multiple range test was applied for significant difference analysis. *p* < 0.05 indicates a significant difference, while *p* > 0.05 indicates no significant difference.

## 4. Conclusions

This study successfully prepared AChE inhibitory peptides from yellowfin tuna pancreas using MUE for the first time. The pancreatic protein hydrolysate with potent AChE inhibitory activity could be obtained under the optimum MUE conditions, including a solid-to-liquid ratio of 1:30 mg/mL, papain dosage of 8053 U/g, ultrasonic power of 253 W, ultrasonic duration of 20 min, and enzymatic hydrolysis time of 3.52 h. The F3 with the strongest AChE inhibitory activity was isolated from pancreatic protein hydrolysate using ultrafiltration and RP-HPLC. Subsequently, a novel AChE inhibitory peptide, LLDF, was identified from the F3 via LC-MS/MS and virtual screening. LLDF exhibited reversible competitive inhibition of AChE, with an IC_50_ value of 18.44 ± 0.24 μM. Molecular docking and molecular dynamics simulations demonstrated that LLDF inhibited AChE activity by forming a stable complex primarily through hydrogen bonding interactions. These findings are significant as they enhance the value of tuna by-products and offer new avenues for AD treatment using marine-derived AChE inhibitory peptides.

## Figures and Tables

**Table 1:** Statistical analysis of the Plackett–Burman design.

Source	Sum of Squares	df	Mean Square	F-Value	*p*-Value
Model	41.96	5	8.39	24.21	0.0007
A-Enzyme dosage	11.87	1	11.87	34.24	0.0011
B-Ultrasonic power	18.10	1	18.10	52.20	0.0004
C-Ultrasonic duration	2.00	1	2.00	5.76	0.0533
D-Enzymatic hydrolysis time	9.99	1	9.99	28.82	0.0017
E-Solid-to-liquid ratio	0.0033	1	0.0033	0.0096	0.9250
Residual	2.08	6	0.3467		
Cor Total	44.04	1			
R^2^					0.9527
R^2^_Adj_					0.9134
CV (%)	1.53				
Adeq Precision	17.02				

**Table 2:** Design and results of RSM with AChE inhibitory activity as response value.

Number	Enzyme Dosage (U/g)	Ultrasonic Power (W)	Enzymatic Hydrolysis Time (h)	AChE Inhibitory Activity (%)
1	9000	250	3.0	33.61
2	8000	225	4.0	34.26
3	8000	250	3.5	42.05
4	8000	250	3.5	42.89
5	7000	250	3.0	33.21
6	7000	250	4.0	33.25
7	9000	225	3.5	32.26
8	8000	250	3.5	42.42
9	8000	275	4.0	36.15
10	9000	250	4.0	34.16
11	7000	225	3.5	32.32
12	8000	250	3.5	42.15
13	8000	225	3.0	33.16
14	8000	250	3.5	42.76
15	9000	275	3.5	34.31
16	7000	275	3.5	31.31
17	8000	275	3.0	36.16

**Table 3:** ANOVA for RSM quadratic model with AChE inhibitory activity as response value.

Source	Sum of Squares	Mean Square	F-Value	*p*-Value
Model	293.06	32.56	82.82	<0.0001
A-Enzyme dosage	2.25	2.25	5.73	0.0479
B-Ultrasonic power	4.40	4.40	11.18	0.0124
C-Enzyme hydrolysis time	0.3570	0.3570	0.9080	0.3724
AB	2.35	2.35	5.97	0.0446
AC	0.0658	0.0658	0.1673	0.6947
BC	0.3086	0.3086	0.7849	0.4051
A^2^	134.05	134.05	340.94	<0.0001
B^2^	76.60	76.60	194.83	<0.0001
C^2^	44.67	44.67	113.63	<0.0001
Residual	2.75	0.3932		
Lack of Fit	2.22	0.7396	5.55	0.0657
Pure Error	0.5334	0.1334		
Cor Total	295.82			
R^2^				0.9907
R^2^_Adj_				0.9787
CV (%)	1.73			
Adeq Precision	22.6319			

## Data Availability

The supporting data for this study are available within the article and Supplementary Materials.

## Supplementary Materials

The following supporting information can be downloaded at: https://www.mdpi.com/article/10.3390/md23020075/s1, Method S1: Identification of AChE inhibitory peptides; Table S1: Design and results of Plackett–Burman with AChE inhibitory activity as response value; Table S2: Energy components of LLDF-AChE (kcal/mol).

## Abbreviations

The following abbreviations are used in this manuscript:

AD	Alzheimer’s disease
AChE	Acetylcholinesterase
ACh	Acetylcholine
CE	Conventional enzymatic hydrolysis
UPE	Ultrasonic pretreatment coupled with enzymatic hydrolysis
MUE	Moderate ultrasonic-assisted enzymatic hydrolysis
DH	Degree of hydrolysis
FT-IR	Fourier transform infrared spectroscop
SDS-PAGE	Sodium dodecyl sulfate-polyacrylamide gel electrophoresis
RSM	Response surface methodology
RMSD	The root mean square deviation
RMSF	Root mean square fluctuation
Rg	Radius of gyration
PCA	Principal component analysis
ATCI	Acetylthiocholine iodide

## References

[1] Dahiya M., Kumar A., Yadav M. (2023). Ameliorative effect of *β*-pinene targeting mitochondrial dysfunction and oxidative stress in Alzheimer’s disease. Alzheimer’s Dement..

[2] Singh Y.P., Kumar H. (2024). Recent advances in medicinal chemistry of memantine against alzheimer’s disease. Chem. Biol. Drug Des..

[3] Babu A., John M., Liji M.J., Maria E., Bhaskar S.J., Binukmar B.K., Sajith A.M., Reddy E.K., Dileep K.V., Sunil K. (2023). Sub-pocket-focused designing of tacrine derivatives as potential acetylcholinesterase inhibitors. Comput. Biol. Med..

[4] Atrahimovich D., Avni D., Khatib S. (2021). Flavonoids-macromolecules interactions in human diseases with focus on alzheimer, atherosclerosis and cancer. Antioxidants.

[5] Cheng L., Shi C., Li X., Matsui T. (2024). Impact of peptide transport and memory function in the brain. Nutrients.

[6] Rafique H., Hu X., Ren T., Dong R., Aadil R.M., Zou L., Sharif M.K., Li L. (2023). Characterization and exploration of the neuroprotective potential of oat-protein-derived peptides in PC12 cells and scopolamine-treated zebrafish. Nutrients.

[7] Wang S., Zheng L., Zhao T., Zhang Q., Liu Y., Sun B., Su G., Zhao M. (2020). Inhibitory effects of walnut (*Juglans regia*) peptides on neuroinflammation and oxidative stress in lipopolysaccharide-induced cognitive impairment mice. J. Agric. Food Chem..

[8] Asen N.D., Aluko R.E. (2022). Acetylcholinesterase and butyrylcholinesterase inhibitory activities of antioxidant peptides obtained from enzymatic pea protein hydrolysates and their ultrafiltration peptide fractions. J. Food Biochem..

[9] Zhao T., Su G., Wang S., Zhang Q., Zhang J., Zheng L., Sun B., Zhao M. (2017). Neuroprotective effects of acetylcholinesterase inhibitory peptides from anchovy (*Coilia mystus*) against glutamate-induced toxicity in PC12 cells. J. Agric. Food Chem..

[10] Zhao T., Zhang C., Zhong S., Chen Q., Liu S., Jiao W., Liu W., Huang L., Zhang Y., Zhang Y. (2022). Synergistic alleviation effects of anchovy hydrolysates-catechin on scopolamine-induced mice memory deficits: The exploration of the potential relationship among gut-brain-axis. Food Funct..

[11] Ulug S.K., Jahandideh F., Wu J. (2021). Novel technologies for the production of bioactive peptides. Trends Food Sci. Technol..

[12] Qian J., Chen D., Zhang Y., Gao X., Xu L., Guan G., Wang F. (2023). Ultrasound-assisted enzymatic protein hydrolysis in food processing: Mechanism and parameters. Foods.

[13] Nadar S.S., Rathod V.K. (2017). Ultrasound assisted intensification of enzyme activity and its properties: A mini-review. World J. Microbiol. Biotechnol..

[14] Chen L., Chen S., Rong Y., Zeng W., Hu Z., Ma X., Feng S. (2024). Identification and evaluation of antioxidant peptides from highland barley distiller’s grains protein hydrolysate assisted by molecular docking. Food Chem..

[15] Hao Y., Xing L., Wang Z., Cai J., Toldrá F., Zhang W. (2023). Study on the anti-inflammatory activity of the porcine bone collagen peptides prepared by ultrasound-assisted enzymatic hydrolysis. Ultrason. Sonochem..

[16] Liu H., Sun H.-N., Zhang M., Mu T.-H., Khan N.M. (2023). Production, identification and characterization of antioxidant peptides from potato protein by energy-divergent and gathered ultrasound assisted enzymatic hydrolysis. Food Chem..

[17] Ding Y., Wang Y., Qu W., Ren X., Lu F., Tian W., Quaisie J., Azam S.M.R., Ma H. (2022). Effect of innovative ultrasonic frequency excitation modes on rice protein: Enzymolysis and structure. LWT-Food Sci. Technol..

[18] Hu H., Wu J., Li-Chan E.C.Y., Zhu L., Zhang F., Xu X., Fan G., Wang L., Huang X., Pan S. (2013). Effects of ultrasound on structural and physical properties of soy protein isolate (SPI) dispersions. Food Hydrocoll..

[19] Wen C., Zhang J., Zhou J., Feng Y., Duan Y., Zhang H., Ma H. (2020). Slit divergent ultrasound pretreatment assisted watermelon seed protein enzymolysis and the antioxidant activity of its hydrolysates in vitro and in vivo. Food Chem..

[20] Wang B., Atungulu G.G., Khir R., Geng J., Ma H., Li Y., Wu B. (2014). Ultrasonic treatment effect on enzymolysis kinetics and activities of ACE-inhibitory peptides from oat-isolated protein. Food Biophys..

[21] Iqbal A., Murtaza A., Marszałek K., Iqbal M.A., Chughtai M.F.J., Hu W., Barba F.J., Bi J., Liu X., Xu X. (2020). Inactivation and structural changes of polyphenol oxidase in quince (*Cydonia oblonga* Miller) juice subjected to ultrasonic treatment. J. Sci. Food Agric..

[22] Wang X., Shen H., Sun X., Yu R., Liu B., Shu X. (2019). Protective effect of pancreatic trypsin from tuna against oxidative damage induced by H_2_O_2_ in insulinoma cells. Oceanol. Limnol. Sin..

[23] Ye M., Jia W., Zhang C., Shen Q., Zhu L., Wang L. (2019). Preparation, identification and molecular docking study of novel osteoblast proliferation-promoting peptides from yak (*Bos grunniens*) bones. RSC Adv..

[24] Han J., Li Y., Cui C., Dong L. (2018). Regulatory mechanism of the pancreas enzyme solution of tuna on the kidney function of type 2 diabetic mice. J. Chin. Inst. Food Sci. Technol..

[25] Aberoumand A., Fazeli A. (2019). Comparison of analysis and the nutritional value of fresh common carp, frozen and southern canned tuna. Potravin. Slovak J. Food Sci..

[26] Wu Y., He Y., Zhao Z., Yu H., Guo Z., Xiao J. (2024). Analysis of nutrient and fatty acid composition of different by-products of yellowfin tuna in South China Sea. Sci. Technol. Food Ind..

[27] Wan X., Li Y., Wang Q., Wang Q., Chen Y., Shu X. (2012). Effects of tuna pancreas enzyme solution on blood glucose and blood lipids in diabetic rats. J. Chin. Inst. Food Sci. Technol..

[28] Mora L., Toldrá F. (2023). Advanced enzymatic hydrolysis of food proteins for the production of bioactive peptides. Curr. Opin. Food Sci..

[29] Karami Z., Akbari-adergani B. (2019). Bioactive food derived peptides: A review on correlation between structure of bioactive peptides and their functional properties. J. Food Sci. Technol..

[30] Bankova L.G., Barrett N.A., Yoshimoto E., Ualiyeva S. (2019). Isolation and quantitative evaluation of brush cells from mouse tracheas. J. Vis. Exp..

[31] Li S., Yang X., Zhang Y., Ma H., Liang Q., Qu W., He R., Zhou C., Mahunu G.K. (2016). Effects of ultrasound and ultrasound assisted alkaline pretreatments on the enzymolysis and structural characteristics of rice protein. Ultrason. Sonochem..

[32] Li Q., Zhang X., Tang S., Mi S., Lu L., Zeng Q., Xia M., Cai Z. (2022). Improved effect of ultrasound-assisted enzymolysis on egg yolk powder: Structural properties, hydration properties and stability characteristics. Food Chem..

[33] Zhao F., Zhai X., Liu X., Lian M., Liang G., Cui J., Dong H., Wang W. (2021). Effects of high-Intensity ultrasound pretreatment on structure, properties, and enzymolysis of walnut protein isolate. Molecules.

[34] Umego E.C., He R., Ren W., Xu H., Ma H. (2021). Ultrasonic-assisted enzymolysis: Principle and applications. Process Biochem..

[35] Rahman M.M., Lamsal B.P. (2021). Ultrasound-assisted extraction and modification of plant-based proteins: Impact on physicochemical, functional, and nutritional properties. Compr. Rev. Food Sci. Food Saf..

[36] Zhang Y., Wang B., Zhou C. (2016). Surface topography, nano-mechanics and secondary structure of wheat gluten pretreated by alternate dual-frequency ultrasound and the correlation to enzymolysis. Ultrason. Sonochem..

[37] Lindsay Rojas M., Hellmeister Trevilin J., Augusto P.E.D. (2016). The ultrasound technology for modifying enzyme activity. Sci. Agropecu..

[38] Stefanović A.B., Jovanović J.R., Balanč B.D., Šekuljica N.Ž., Tanasković S.M.J., Dojčinović M.B., Knežević-Jugović Z.D. (2018). Influence of ultrasound probe treatment time and protease type on functional and physicochemical characteristics of egg white protein hydrolysates. Poult. Sci..

[39] Lan M., Li W., Chang C., Liu L., Li P., Pan X., Ma X., He C., Jiao Y. (2020). Enhancement on enzymolysis of pigskin with ultrasonic assistance. Bioengineered.

[40] Wang S., Wang J., Xue F., Li C. (2019). Effects of heating or ultrasound treatment on the enzymolysis and the structure characterization of hempseed protein isolates. J. Food Sci. Technol..

[41] Yu L., Sun J., Liu S., Bi J., Zhang C., Yang Q. (2012). Ultrasonic-assisted enzymolysis to improve the antioxidant activities of peanut (*Arachin conarachin* L.) antioxidant hydrolysate. Int. J. Mol. Sci..

[42] Hu X., Liu J., Li J., Song Y., Chen S., Zhou S., Yang X. (2022). Preparation, purification, and identification of novel antioxidant peptides derived from *Gracilariopsis lemaneiformis* protein hydrolysates. Front. Nutr..

[43] Wang R., Yun J., Wu S., Bi Y., Zhao F. (2022). Optimisation and characterisation of novel angiotensin-converting enzyme inhibitory peptides prepared by double enzymatic hydrolysis from *Agaricus bisporus* scraps. Foods.

[44] Zhang H., Zhang Z., He D., Li S., Xu Y. (2022). Optimization of enzymatic hydrolysis of *Perilla* meal protein for hydrolysate with high hydrolysis degree and antioxidant activity. Molecules.

[45] Kusumah J., Real Hernandez L.M., Gonzalez de Mejia E. (2020). Antioxidant potential of mung bean (*Vigna radiata*) albumin peptides produced by enzymatic hydrolysis analyzed by biochemical and In silico methods. Foods.

[46] Mir Mohammad Sadeghi H., Mohammadian N., Mohammadian H., Moazen F., Pakdel M., Jahanian-Najafabadi A. (2020). Optimization of solvent media to solubilize TEV protease using response surface method. Res. Pharm. Sci..

[47] Antonow M., Franco C., Prado W., Beckenkamp A., Silveira G., Buffon A., Guterres S., Pohlmann A. (2017). Arginylglycylaspartic acid-surface-functionalized doxorubicin-loaded lipid-core nanocapsules as a strategy to target alpha(V) beta(3) integrin expressed on tumor cells. Nanomaterials.

[48] Yu H., Xian M., Qu C., Peng P., Yongo E., Guo Z., Du Z., Xiao J. (2024). Novel se-enriched *α*-glucosidase inhibitory peptide derived from tuna dark meat: Preparation, identification and effects on IR-HepG2 cells. Food Biosci..

[49] Asen N.D., Okagu O.D., Udenigwe C.C., Aluko R.E. (2022). In vitro inhibition of acetylcholinesterase activity by yellow field pea (*Pisum sativum*) protein-derived peptides as revealed by kinetics and molecular docking. Front. Nutr..

[50] Malomo S.A., Aluko R.E. (2019). Kinetics of acetylcholinesterase inhibition by hemp seed protein-derived peptides. J. Food Biochem..

[51] Su G., Chen J., Huang L., Zhao M., Huang Q., Zhang J., Zeng X., Zhang Y., Deng L., Zhao T. (2024). Effects of walnut seed coat polyphenols on walnut protein hydrolysates: Structural alterations, hydrolysis efficiency, and acetylcholinesterase inhibitory capacity. Food Chem..

[52] Xu B., Xu X., Zhang C. (2017). Synthesis and protective effect of new ligustrazine-vanillic acid derivatives against CoCl2-induced neurotoxicity in differentiated PC12 cells. Chem. Cent. J..

[53] Yu Z., Wu S., Zhao W., Ding L., Fan Y., Shiuan D., Liu J., Chen F. (2018). Anti-Alzheimers activity and molecular mechanism of albumin-derived peptides against AChE and BChE. Food Funct..

[54] Asen N.D., Udenigwe C.C., Aluko R.E. (2023). Quantitative structure–activity relationship modeling of pea protein-derived acetylcholinesterase and butyrylcholinesterase inhibitory peptides. J. Agric. Food Chem..

[55] Liu W., Yang W., Li X., Qi D., Chen H., Liu H., Yu S., Wang G., Liu Y. (2022). Evaluating the properties of ginger protease-degraded collagen hydrolysate and identifying the cleavage site of ginger protease by using an integrated strategy and LC-MS technology. Molecules.

[56] Liu X., Jiang L., Li L., Lu F., Liu F. (2023). Bionics design of affinity peptide inhibitors for SARS-CoV-2 RBD to block SARS-CoV-2 RBD-ACE2 interactions. Heliyon.

[57] Alshammari A., Alasmari A.F., Alharbi M., Ali N., Muhseen Z.T., Ashfaq U.A., Ud-din M., Ullah A., Arshad M., Ahmad S. (2022). Novel chimeric vaccine candidate development against *Leptotrichia buccalis*. Int. J. Environ. Res. Public Health.

[58] Yu Z., Ji H., Shen J., Kan R., Zhao W., Li J., Ding L., Liu J. (2020). Identification and molecular docking study of fish roe-derived peptides as potent BACE 1, AChE, and BChE inhibitors. Food Funct..

[59] Dastan D., Fasihi K., Ebadi A. (2020). From venom to AChE Inhibitor: Design, molecular modeling, and synthesis of a peptidic inhibitor of AChE. Int. J. Pept. Res. Ther..

[60] Sanchis I., Spinelli R., Aschemacher N., Humpola M.V., Siano A. (2020). Acetylcholinesterase inhibitory activity of a naturally occurring peptide isolated from *Boana pulchella* (Anura: Hylidae) and its analogs. Amino Acids.

[61] Zhou H., Liao J., Ou J., Lin J., Zheng J., Li Y., Ou S., Liu F. (2022). Bioassay-guided isolation of Fenghuang Dancong tea constituents with *α*-glucosidase inhibition activities. Front. Nutr..

[62] Lopez S.M.M., Aguilar J.S., Fernandez J.B.B., Lao A.G.J., Estrella M.R.R., Devanadera M.K.P., Ramones C.M.V., Villaraza A.J.L., Guevarra Jr L.A., Santiago-Bautista M.R. (2021). Neuroactive venom compounds obtained from *Phlogiellus bundokalbo* as potential leads for neurodegenerative diseases: Insights on their acetylcholinesterase and beta-secretase inhibitory activities in vitro. J. Venom. Anim. Toxins Incl. Trop. Dis..

[63] Qiu J., Xu M., Ren R., Zhao Y., Liu L., Li X., Zhu X., Ji H., Geng Y., Huang X. (2024). Identification, inhibition modes, and molecular docking of ACE inhibitory peptides derived from Cheddar cheese. LWT-Food Sci. Technol..

[64] Istyastono E.P., Riswanto F.D.O., Yuniarti N., Prasasty V.D., Mungkasi S. (2022). Pyplif Hippos and receptor ensemble docking increase the prediction accuracy of the structure-based virtual screening protocol targeting acetylcholinesterase. Molecules.

[65] Sajal H., Patil S.M., Raj R., Shbeer A.M., Ageel M., Ramu R. (2022). Computer-aided screening of phytoconstituents from *Ocimum tenuiflorum* against diabetes mellitus targeting DPP4 Inhibition: A combination of molecular docking, molecular dynamics, and pharmacokinetics approaches. Molecules.

[66] Yang R., Zhao G., Zhang L., Xia Y., Yu H., Yan B., Cheng B. (2022). Identification of potential extracellular signal-regulated protein kinase 2 inhibitors based on multiple virtual screening strategies. Front. Pharmacol..

[67] Razzaghi-Asl N., Mirzayi S., Mahnam K., Adhami V., Sepehri S. (2022). In silico screening and molecular dynamics simulations toward new human papillomavirus 16 type inhibitors. Res. Pharm. Sci..

[68] Khan M.A., Al Mamun Khan M.A., Mahfuz A.M.U.B., Sanjana J.M., Ahsan A., Gupta D.R., Hoque M.N., Islam T. (2022). Highly potent natural fungicides identified in silico against the cereal killer fungus *Magnaporthe oryzae*. Sci. Rep..

[69] Kulkarni A.M., Parate S., Lee G., Kim Y., Jung T.S., Lee K.W., Ha M.W. (2022). Computational simulations highlight the IL2Rα binding potential of polyphenol stilbenes from fenugreek. Molecules.

[70] Jeevana R., Kavitha A.P., Abi T.G., Sajith P.K., Varughese J.K., Aravindakshan K.K. (2022). Targeting COVID-19 pandemic: In silico evaluation of 2-hydroxy-1, 2-diphenylethanone N(4)-methyl-N(4)-phenylthiosemicarbazone as a potential inhibitor of SARS-CoV-2. Struct. Chem..

[71] Hong C., Zhu J.-Q., Zhao Y.-M., Ma H. (2022). Effects of dual-frequency slit ultrasound on the enzymolysis of high-concentration hydrolyzed feather meal: Biological activities and structural characteristics of hydrolysates. Ultrason. Sonochem..

[72] Liang Y., Guo Y., Zheng Y., Liu S., Cheng T., Zhou L., Guo Z. (2022). Effects of high-pressure homogenization on physicochemical and functional properties of enzymatic hydrolyzed soybean protein concentrate. Front. Nutr..

[73] Wu K., Liao Y.-T., Liu C.-H., Yu J. (2014). Liver cancer cells: Targeting and prolonged-release drug carriers consisting of mesoporous silica nanoparticles and alginate microspheres. Int. J. Nanomed..

[74] Moreira T.F.M., Pessoa L.G.A., Seixas F.A.V., Ineu R.P., Gonçalves O.H., Leimann F.V., Ribeiro R.P. (2022). Chemometric evaluation of enzymatic hydrolysis in the production of fish protein hydrolysates with acetylcholinesterase inhibitory activity. Food Chem..

