# Supplementary material for: Preparation of Acetylcholinesterase Inhibitory Peptides from Yellowfin Tuna Pancreas Using Moderate Ultrasound-Assisted Enzymatic Hydrolysis
Source: Mar Drugs. 2025 Feb 9;23(2):75. doi: 10.3390/md23020075 (PMC11857449; doi:10.3390/md23020075)
Supplement: Supplementary file 1 [file marinedrugs-23-00075-s001.zip › marinedrugs-3447495-supplementary.pdf]

Supplementary Information

Identification of AChE inhibitory peptides

Peptide analysis was performed using an Easy-nLC 1200 nanoflow UPLC system (Thermo Fisher Scientific, USA) coupled to a Q Exactive™ Quadrupole-Orbitrap™ mass spectrometer (Thermo Fisher Scientific, USA). Chromatographic separation was accomplished on an Acclaim PepMap RPLC C18 column (150 μm × 150 mm, 1.9 μm, Dr Maisch GmbH, Germany) at a flow rate of 600 nL min−1. The mobile phases consisted of (A) ultrapure water with 0.1% FA and (B) 80% ACN with 0.1% FA. The gradient elution analysis was conducted at 4–8% (B) for 0–2 min, 8–28% (B) for 2–45 min, 28–40% (B) for 55–56 min, 40-95% (B) for 55–56 min, and 95% (B) for 56–66 min. The MS precursor m/z range was set at 100−1500, and the resolution was set at 70,000, the capillary temperature was 270℃, and the spray voltage was 2.2 kV. PEAKS Studio 10.6 (Bioinformatics Solutions Inc. Waterloo, Canada) de novo peptide sequencing tool was used to analyze the MS/MS data. The following settings were chosen for variable modifications: oxidation (M), and acetylation (protein N-term), carboxy-domethylation (C). Precursor and fragment mass tolerance were set to 20 ppm and 0.02 Da, respectively.

Table S1 Design and results of Plackett–Burman with AChE inhibitory activity as response value

No.	Enzyme dosage (U/g)	Ultrasonic power (W)	Ultrasonic duration (min)	Enzymatic hydrolysis time (h)	Solid-to-liquid (mg/mL)	AChE inhibitory activity (%)
1	8000	250	25	3	1:30	41.49
2	8000	200	25	4	1:30	38.50
3	8000	250	20	3	1:30	41.49
4	8000	200	20	3	1:20	38.99
5	6000	200	20	3	1:30	37.11
6	8000	200	25	4	1:20	37.41
7	6000	250	25	3	1:20	39.98
8	6000	250	25	4	1:30	38.66
9	6000	250	20	4	1:20	37.66
10	6000	200	20	4	1:30	34.22
11	8000	250	20	4	1:20	39.65
12	6000	200	25	3	1:20	37.97

Table S2 Energy components of LLDF-AChE (kcal/mol)

	**Energy**
$\Delta G_{VDWAALS}$	-41.04
ΔG_{EEL}	90.25
ΔG_{EGB}	-62.03
ΔG_{ESURF}	-5.98
ΔG_{GGAS}	49.21
ΔG_{GSOLV}	-68.01
ΔG_{TOTAL}	-18.8

* VDWAALS: van der Waals energy;
EEL: Electrostatic energy;
EGB: Polar solvation energy;
ESURF: Non-polar solvation energy;
GGAS: Total gas phase free energy, VDWAALS + EEL;
GSOLV: Total solvation free energy, EGB + ESURF;
TOTAL: Total free energy, GSOLV + GGAS
